# 27.4 STRUCTURAL, FUNCTIONAL, AND BEHAVIORAL INSIGHTS OF DOPAMINE DYSFUNCTION REVEALED BY A DELETION IN SLC6A3

**DOI:** 10.1093/schbul/sby014.114

**Published:** 2018-04-01

**Authors:** Aurelio Galli

**Affiliations:** Vanderbilt University

## Abstract

**Background:**

The human dopamine (DA) transporter (hDAT) mediates clearance of DA. Genetic variants in hDAT are associated to neuropsychiatric disorders. We investigated the structural and behavioral bases of an in-frame deletion in hDAT at N336 (∆N336) associated with neuropsychiatric disorders.

**Methods:**

This study bridges structural biology, molecular neuroscience and organism physiology culminating in a mechanistic model that relates precise alteration in a transport cycle with behavioral manifestations.

**Results:**

We uncovered a previously unobserved conformation of the intracellular gate of the transporter promoted by ∆N336, representing likely the rate limiting step of the transport process. This state is defined by a “half-open and inward facing” state (HOIF) of the intracellular gate that leads to DA dysfunction. The HOIF state is regulated by a network of interactions conserved phylogenetically, as we observed it both in hDAT and in its bacterial homolog leucine transporter. We demonstrated these dysfunctions in brains of Drosophila melanogaster expressing hDAT ∆N336. These flies are hyperactive and display increased fear and impaired social interactions, traits associated with neuropsychiatric disorders.

**Discussion:**

Here, we describe how a genetic variation causes DA dysfunction. In this study different techniques and discoveries came together to detail in a translational effort how rare variants in plasma membrane proteins affect complex behavior.

